# Amelioration of Endotoxin-Induced Acute Lung Injury and Alveolar Epithelial Cells Apoptosis by Simvastatin Is Associated with Up-Regulation of Survivin/NF-kB/p65 Pathway

**DOI:** 10.3390/ijms23052596

**Published:** 2022-02-26

**Authors:** Lana Nežić, Ljiljana Amidžić, Ranko Škrbić, Radoslav Gajanin, Danijela Mandić, Jelena Dumanović, Zoran Milovanović, Vesna Jaćević

**Affiliations:** 1 Department of Pharmacology, Toxicology and Clinical Pharmacology, Faculty of Medicine, University of Banja Luka, 14 Save Mrkalja St., 78000 Banja Luka, Bosnia and Herzegovina; lana.nezic@med.unibl.org (L.N.); ranko.skrbic@med.unibl.org (R.Š.); 2 Center for Biomedical Research, Faculty of Medicine, University of Banja Luka, 14 Save Mrkalja St., 78000 Banja Luka, Bosnia and Herzegovina; ljiljana.amidzic@med.unibl.org; 3 Institute of Pathology, University Clinical Center of Republic of Srpska, Faculty of Medicine, University of Banja Luka, 12 Beba St., 78000 Banja Luka, Bosnia and Herzegovina; radoslav.gajanin@med.unibl.org; 4 Department of Internal Medicine, School of Medicine, University of Banja Luka, 14 Save Mrkalja St., 78000 Banja Luka, Bosnia and Herzegovina; danijela.mandic@kc-bl.com; 5 Faculty of Chemistry, University of Belgrade, 16 Studenski trg St., 11000 Belgrade, Serbia; dumanovicjelena@gmail.com; 6 Medical Faculty of the Military Medical Academy, University of Defence in Belgrade, 17 Crnotravska St., 11000 Belgrade, Serbia; 7 Special Police Unit, Police Department of the City of Belgrade, Ministry of Interior, 12/A Trebevićka St., 11030 Belgrade, Serbia; tinahols41@gmail.com; 8 Department for Experimental Toxicology and Pharmacology, National Poison Control Centre, Military Medical Academy, 11 Crnotravska St., 11000 Belgrade, Serbia; 9 Department of Chemistry, Faculty of Science, University of Hradec Kralove, 62 Rokitanského St., 500 03 Hradec Králové, Czech Republic

**Keywords:** simvastatin, alveolar epithelial cells, apoptosis, survivin, NF-kB/p65

## Abstract

Disruption of the alveolar–endothelial barrier caused by inflammation leads to the progression of septic acute lung injury (ALI). In the present study, we investigated the beneficial effects of simvastatin on the endotoxin lipopolysaccharide (LPS)-induced ALI and its related mechanisms. A model of ALI was induced within experimental sepsis developed by intraperitoneal injection of a single non-lethal LPS dose after short-term simvastatin pretreatment (10–40 mg/kg orally). The severity of the lung tissue inflammatory injury was expressed as pulmonary damage scores (PDS). Alveolar epithelial cell apoptosis was confirmed by TUNEL assay (DNA fragmentation) and expressed as an apoptotic index (AI), and immunohistochemically for cleaved caspase-3, cytochrome C, and anti-apoptotic Bcl-xL, an inhibitor of apoptosis, survivin, and transcriptional factor, NF-kB/p65. Severe inflammatory injury of pulmonary parenchyma (PDS 3.33 ± 0.48) was developed after the LPS challenge, whereas simvastatin significantly and dose-dependently protected lung histology after LPS (*p* < 0.01). Simvastatin in a dose of 40 mg/kg showed the most significant effects in amelioration alveolar epithelial cells apoptosis, demonstrating this as a marked decrease of AI (*p* < 0.01 vs. LPS), cytochrome C, and cleaved caspase-3 expression. Furthermore, simvastatin significantly enhanced the expression of Bcl-xL and survivin. Finally, the expression of survivin and its regulator NF-kB/p65 in the alveolar epithelium was in strong positive correlation across the groups. Simvastatin could play a protective role against LPS-induced ALI and apoptosis of the alveolar–endothelial barrier. Taken together, these effects were seemingly mediated by inhibition of caspase 3 and cytochrome C, a finding that might be associated with the up-regulation of cell-survival survivin/NF-kB/p65 pathway and Bcl-xL.

## 1. Introduction

In spite of extensive research on treating acute respiratory distress syndrome (ARDS), one of the severe complications of acute lung injury (ALI), the overall mortality remains high. Pathogenesis of ALI involves exaggerated pulmonary inflammation, leading to an impairment of the alveolar–capillary barrier, structural disarrangement of surfactant, and deterioration of gas exchange [1].

Lipopolysaccharide (LPS), an endotoxin and a key component of the Gram-negative bacterial cell wall, plays a complex role in lung inflammation and its systemic or intranasal administration has been widely used to induce pulmonary inflammation in an animal model of ALI [2,3]. Endotoxin binds Toll-like receptor (TLR)-4 on macrophages, endothelium, airway, and alveolar epithelium (type I and II), leading to the production of chemokines and pro-inflammatory cascade, such as tumor necrosis factor (TNF)-α and interleukin-1β, which might be crucial in the ALI pathogenesis [2,3]. Moreover, apoptosis is one of the critical factors in the development of ALI induced by LPS and partially understood in ALI associated with SARS-CoV-2 infection [1,4]. Binding TLR-4, LPS activates pro-apoptotic signals in different types of cells or triggers augmentation of intracellular reactive oxygen (ROS) and nitrogen species that lead to mitochondrion-dependent apoptosis of alveolar epithelial cells [3,5]. Pleiotropic cytokine TNF-α can also initiate cell-survival signaling through anti-apoptotic molecules Bcl-2 and inhibitors of apoptosis [6]. 

Therefore, many potential therapeutic agents are investigated to target LPS-induced apoptosis in pulmonary parenchymas, such as geraniol, which dramatically reduced lung injury, inhibited pro-apoptotic Bax and caspase-3 expression, and promoted an anti-apoptotic protein of the Bcl-2 family [7]. Additionally, partial inhibition of the mevalonate pathway by targeting geranylgeranyl pyrophosphate synthase large subunit 1 (GGPPS1), was effective in treating LPS-induced ALI by suppressing the NLRP3 inflammasome and apoptosis of alveolar epithelial cells [8].

Survivin is a member of the inhibitor of apoptosis proteins (IAP) that either directly or indirectly interfere with the function of executor caspases of apoptosis. One of the main transcriptional factors, NF-kB up-regulates survivin expression and initiates an anti-apoptotic signaling pathway [9,10]. In spite of a confirmed variety of roles attributed to survivin in embryonic organ and tumor biology, the function of this protein in normal adult tissue remains unclear [10]. Accumulating evidence suggests that survivin is involved and considered as the key mediator of cytoprotection in ALI induced by hyperoxia [9], bleomycin [11], and HCl aspiration in silymarin treatment [12].

Pleiotropic effects of lipid-lowering drugs—statins—related to their anti-inflammatory properties, have been confirmed to increase survival and prevent skin injury in local inflammation [13] and lung injuries in sepsis induced by LPS or cecal ligation and puncture (CLP) [14,15]. These effects are explained by the mechanism of action of statins in terms of the inhibition of hydroxy-3-methylglutaryl-CoA (HMG-CoA) reductase and mevalonate pathway, and the subsequent blockade of activation of downstream substrates involved in several cellular processes, such as apoptosis or cell survival [16,17]. One previous study showed that simvastatin improved endothelial permeability and mitigated sepsis-induced ALI, oxidative stress, and apoptosis by inhibiting the Bax, caspase-3, and toll-like receptor 4 (TLR4)/NF-kB signaling pathway [15]. A recent study demonstrated that pravastatin ameliorates sepsis-induced ALI, improves the alveolar endothelial barrier, and inhibits apoptosis in pulmonary parenchyma through regulating the caveolin-1/eNOS signaling pathway [18].

In our previous research of multi-organ dysfunction syndrome in LPS-induced sepsis, we have shown that simvastatin pretreatment improved survival and significantly suppressed the over-production of pro-inflammatory cytokines, TNF-α, and interleukin (IL)-1β and IL-6 [19]. The results demonstrated that simvastatin pretreatment prevented multi-organ injuries induced by LPS, and showed potential in cell protection of cardiomyocytes, hepatocytes, spleen lymphocytes, and renal tubular cells against apoptosis [20,21,22]. The main findings revealed that simvastatin attenuated cleaved caspase-3 expression along with reduction of apoptotic cell death, and concurrently increased anti-apoptotic molecules, all of which partly explain the cell-protective effects of simvastatin in response to LPS-induced tissue injury [20,21,22]. Whether simvastatin attenuates inflammation and down-regulates apoptosis in pulmonary parenchyma in LPS-induced ALI, however, remains unclear. Therefore, the present study was designed to assess if simvastatin inhibits apoptotic cell death of alveolar epithelial cells, and to identify if these cell-protective effects involve the activation of Bcl-xL and survivin/NF-kB/p65 pathway.

## 2. Results

### 2.1. Effects of Simvastatin on Lung Histopathology in LPS-Induced Inflammation

**Figure 1:**
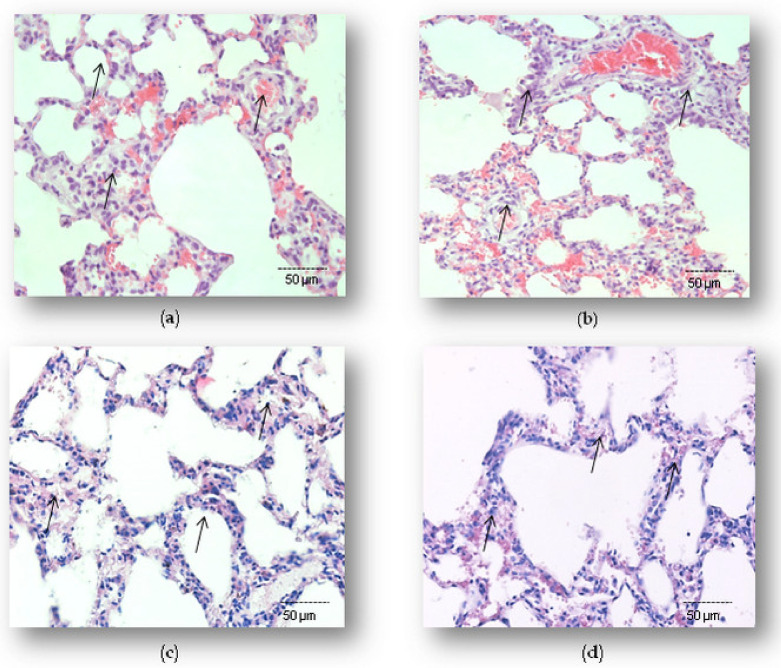
Tissue-protective effects of simvastatin against LPS-induced lung inflammatory injury. Hematoxylin and Eosin (H&E) staining (200×) of pulmonary parenchyma. (**a**) Lung histology of control animals, (**b**) lung histology after challenge with LPS only, (**c**) lung tissues from the group with 20 mg/kg of simvastatin, and (**d**) 40 mg/kg of simvastatin pretreatment prior to LPS. Histopathological analysis revealed decreased lung inflammatory injury in both simvastatin-treated groups, while severe alterations persisted only in the LPS group.

Hematoxylin-eosin staining of the selected tissue sections, and determined pulmonary damage scores (PDSs), indicated the extent of the LPS-induced injury and effects of simvastatin of lung histology. Microscopic examination of the control group showed lung histology (Figure 1a) with normal alveoli and interstitial tissue, pulmonary blood capillaries, and intrapulmonary bronchioles. 

Not surprisingly, a significant lung inflammatory injury was obvious in the animals challenged with LPS only. The lung tissue in the LPS group showed severe inflammatory reactions, including diffuse alveolar capillary congestion, massive focal hemorrhage without tissue necrosis, destructions of or alveolar wall thickening, intensive edema, and diffuse areas of inflammatory cell infiltration into the alveolar space and interstitium resulting in PDS of 3.33 ± 0.48 (*p* < 0.001 vs. control) (Figure 1b and Table 1). Pretreatment with simvastatin (10, 20, and 40 mg/kg) markedly alleviated these histopathological changes induced by LPS (Figure 1c,d). Lung inflammatory injury in the group that received 20 mg/kg of simvastatin was less intensive in comparison to the 40 mg/kg of simvastatin group, where the histology remained mostly unchanged with only rare alveoli filled with desquamated epithelial cells, focal interstitial edema, congestion, and perivascular inflammatory infiltrate in the interstitium. Accordingly, PDSs were significantly prevented by pretreatment with 20 mg/kg of simvastatin and 40 mg/kg of simvastatin (*p* < 0.05 vs. control, *p* < 0.01 vs. LPS group, for both doses of simvastatin). Semiquantitative assessment of lung tissue inflammatory injuries revealed that simvastatin significantly ameliorated LPS-induced histopathological changes in a dose-dependent manner (Table 1).

### 2.2. Simvastatin Attenuated Cytochrome C and Cleaved Caspase-3 Expression and LPS-Induced Apoptosis of Alveolar Epithelial Cells

**Figure 2:**
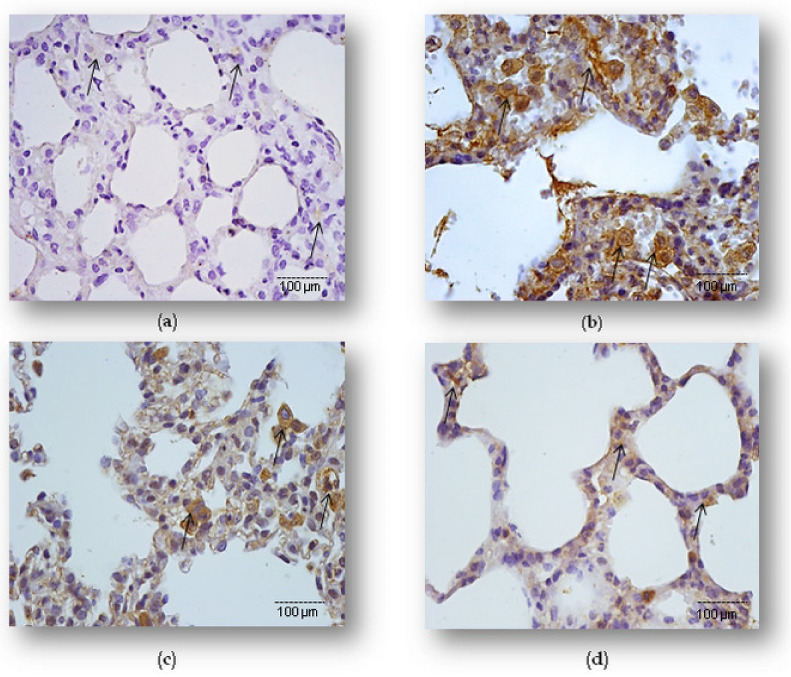
Simvastatin inhibited the apoptosis of alveolar epithelial cells in LPS-induced inflammation by down-regulating cleaved caspase-3 expression (arrows) mainly in the alveolar epithelial cells. Representative images of pulmonary parenchyma that were challenged with LPS or pretreated with 20 mg/kg of simvastatin or 40 mg/kg of simvastatin, respectively, prior to LPS, assessed by immunohistochemistry (400×). (**a**) Control group. (**b**) Intense cytoplasmic staining of cleaved caspase 3 in alveolar epithelium, macrophages, and endothelial cells in the LPS group. Marked reduction of apoptotic alveolar epithelial cells in the groups pretreated with 20 mg/kg of simvastatin (**c**) and 40 mg/kg of simvastatin prior to LPS (**d**).

**Figure 3:**
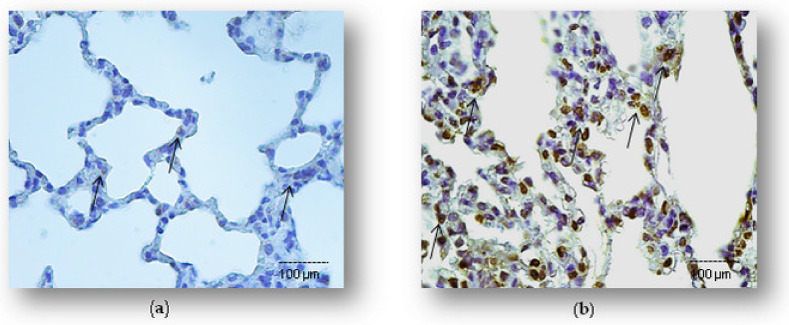

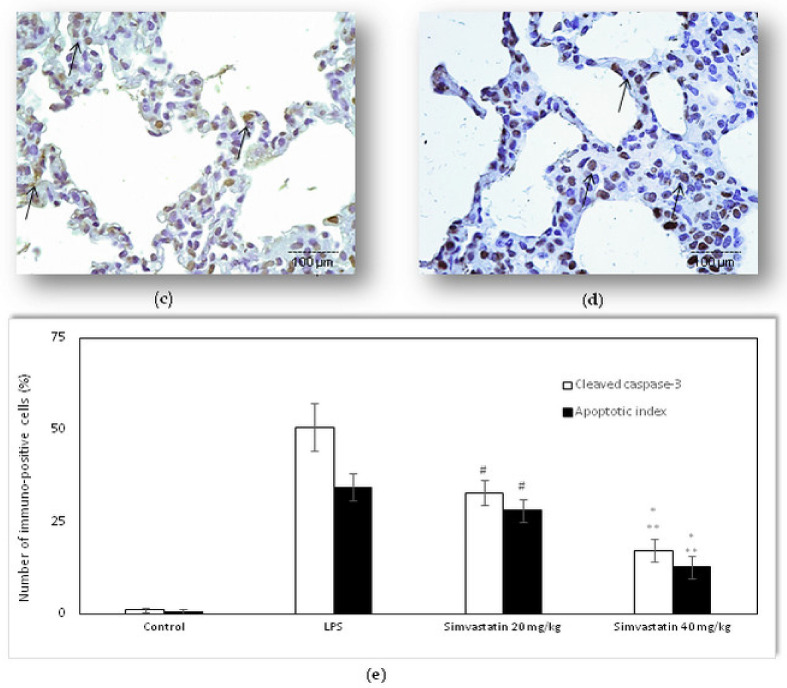
TUNEL assay (400×) of simvastatin effects on alveolar epithelial cells apoptosis in inflammatory injury induced by LPS (**a**–**d**). The apoptotic indices (AIs) refer to the relative number of TUNEL-positive alveolar epithelial cells (shown as brown-stained nuclei, and with arrows). AI (shown as white columns) is markedly increased in LPS group (**b**) while in groups pretreated with 20 mg/kg of simvastatin (**c**) and 40 mg/kg simvastatin prior to LPS (**d**), it decreased significantly and in a dose-dependent manner. Semiquantitative analysis of apoptotic cells counted in the immunohistochemically stained lung tissue for cleaved caspase-3 and corresponding frequencies of TUNEL-positive alveolar epithelial cells expressed as AIs (^#^ *p* < 0.05 vs. LPS group, * *p* < 0.05 vs. 20 mg/kg of simvastatin group, ** *p* < 0.01 vs. LPS group) (**e**).

Based on the results on decreased severity of lung inflammatory injury, we speculated that simvastatin might provide tissue protection to LPS-induced ALI through attenuation of cell apoptosis of pulmonary parenchyma. As illustrated in Figure 2 and Figure 3, compared with the control, the LPS group had a significantly up-regulated expression of cleaved caspase-3 of alveolar epithelial cells, macrophages, and endothelium as well as a substantially increased apoptotic index (AI) (34.5 ± 3.8%, *p* < 0.01, respectively). Pretreatment with 40 mg/kg of simvastatin showed markedly lower incidence of cleaved caspase-3 expressions in alveolar epithelial cells than those administered 20 mg/kg of simvastatin (17.2 ± 3.0%, and 32.9 ± 3.5%, *p* < 0.05), and significantly decreased compared to the LPS group (*p* < 0.01, *p* < 0.05, respectively) (Figure 2b–d). Cleaved caspase-3 is an executioner caspase, best known for its enzymatic function at the end of the inner apoptotic cascade. Additionally, to further confirm the anti-apoptotic effects of simvastatin, we performed a TUNEL analysis. Accordingly, to cleaved caspase-3 results, simvastatin decreased Ais, which are observed as a significantly decreased number of mostly TUNEL-positive alveolar epithelial cells (Figure 3c,d). The extent of apoptosis in pulmonary parenchyma, expressed as the AIs (%), was obviously reduced and seemingly in dose-dependent manner in the 20 mg/kg of simvastatin (AI = 28.0 ± 3.1%) and 40 mg/kg of simvastatin (AI = 12.7 ± 3.0%) groups with respect to the LPS group (*p* < 0.05 and *p* < 0.01, respectively) (Figure 3e).

In the comparison of apoptotic death detected by immunohistochemistry and the TUNEL assay, it is important to emphasize that cleaved caspase-3 expression is confirmed as predominantly brown cytoplasmic staining. Apoptotic activity of cleaved caspase-3 results in the blebbing and condensing of cells that ultimately leads to cell death, but also means pro-apoptotic cells without chromatin condensation and with preserved cellular morphology. Therefore, the results of the apoptosis frequency slightly differ in the total number of cleaved caspase-3 positive cells and TUNEL-positive cells (Figure 3e). These findings are supported by significantly positive correlation between cleaved caspase-3 and TUNEL positive cells in the LPS group (R^2^ = 0.52, *p* < 0.05), 20 mg/kg of simvastatin group (R^2^ = 0.63, *p* < 0.05), and 40 mg/kg of simvastatin group (R^2^ = 0.69, *p* < 0.05).

**Figure 4:**
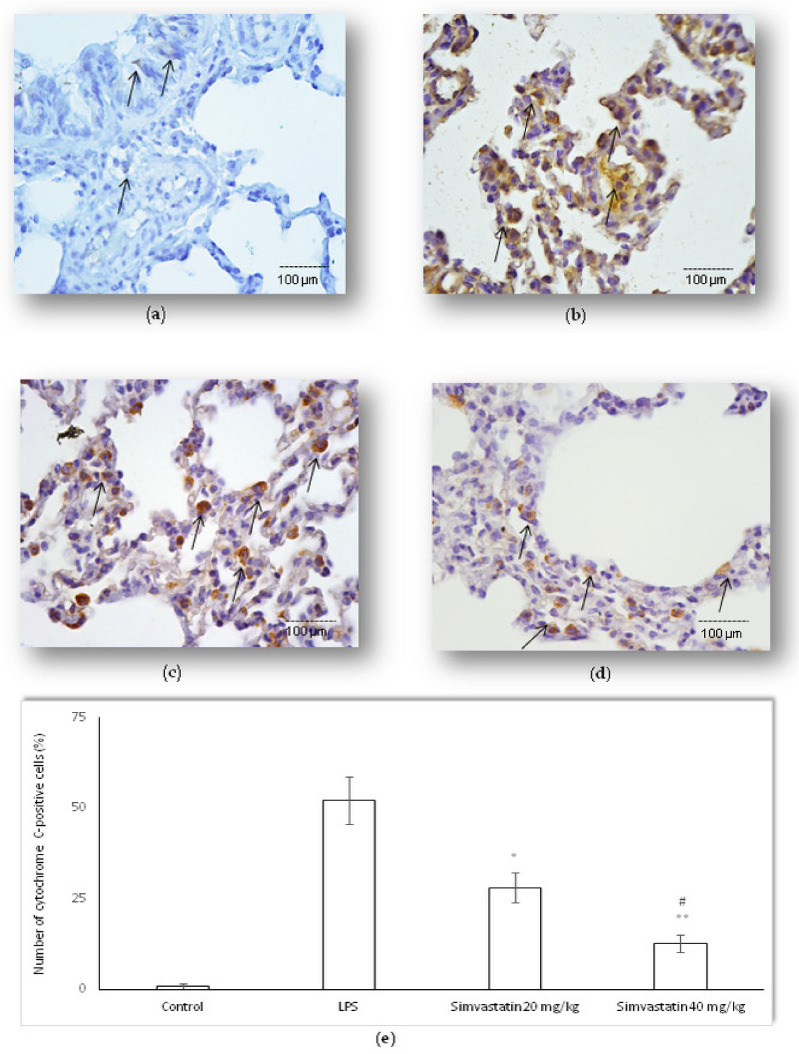
Simvastatin attenuated cytochrome C expression in alveolar epithelial cells in LPS-induced inflammation. Representative images of pulmonary parenchyma that were challenged with LPS or pretreated with 20 mg/kg of simvastatin or 40 mg/kg of simvastatin, respectively, prior to LPS, assessed by immunohistochemistry (400×). (**a**) Control group. (**b**) Intense cytoplasmic staining of cytochrome C (arrows) in alveolar epithelium, macrophages, and endothelial cells in the LPS group. Marked reduction of cytochrome C-positive cells in the groups pretreated with 20 mg/kg of simvastatin (**c**) and 40 mg/kg of simvastatin prior to LPS (**d**). Semiquantitative analysis of cytochrome C-positive cells in pulmonary parenchyma assessed in the immunohistochemically stained sections in the lung tissue (* *p* < 0.05 vs. LPS group, ** *p* < 0.01 vs. LPS group, # *p* < 0.05 vs. 20 mg/kg of simvastatin) (**e**).

**Figure 5:**
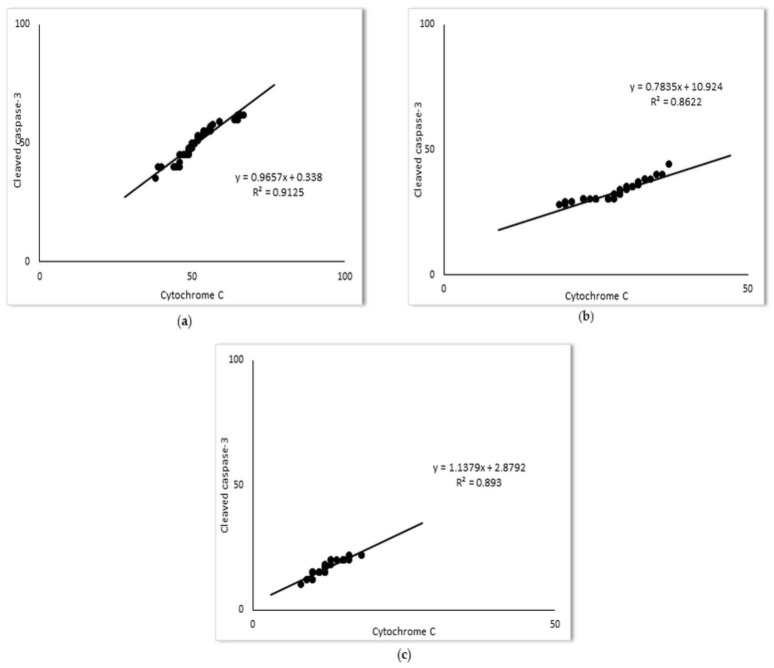
The correlations for cytochrome C and cleaved-caspase-3 in alveolar epithelial cells are shown: (**a**) LPS group, (**b**) 20 mg/kg of simvastatin, (**c**) 40 mg/kg of simvastatin. The number of cytochrome C and cleaved caspase-3 and immunopositive alveolar epithelial cells was quantified across ten non-successive fields per sample of lung tissue from the LPS, 20 mg/kg of simvastatin, and 40 mg/kg of simvastatin groups, using ImageJ software 1.50. The correlation analysis and level of significance were calculated by using Pearson’s correlation coefficient.

Mitochondrial regulation of apoptosis is mediated through the release of cytochrome C in the cytoplasm, and ultimately caspases activation. In the present study, a marked increase of cytochrome C expression (52.1 ± 6.5%) was detected upon LPS administration. As shown in Figure 4, simvastatin pretreatment inhibited LPS-induced cytochrome C up-regulation (*p* < 0.05 vs. LPS with 20 mg/kg of simvastatin group and *p* < 0.01 for the 40 mg/kg of simvastatin group). The anti-apoptotic effects of simvastatin are supported with strong positive correlations of cleaved caspases-3 and cytochrome C expression across the groups (R^2^ = 0.91, *p* < 0.01 in the LPS group, R^2^ = 0.86, in the 20 mg/kg of simvastatin group, and R^2^ = 0.89 in the 40 mg/kg of simvastatin group, *p* < 0.01, respectively) (Figure 5).

### 2.3. Simvastatin Up-Regulated Bcl-xL Expression in Pulmonary Parenchyma in LPS-Induced Inflammatory Injury

**Figure 6:**
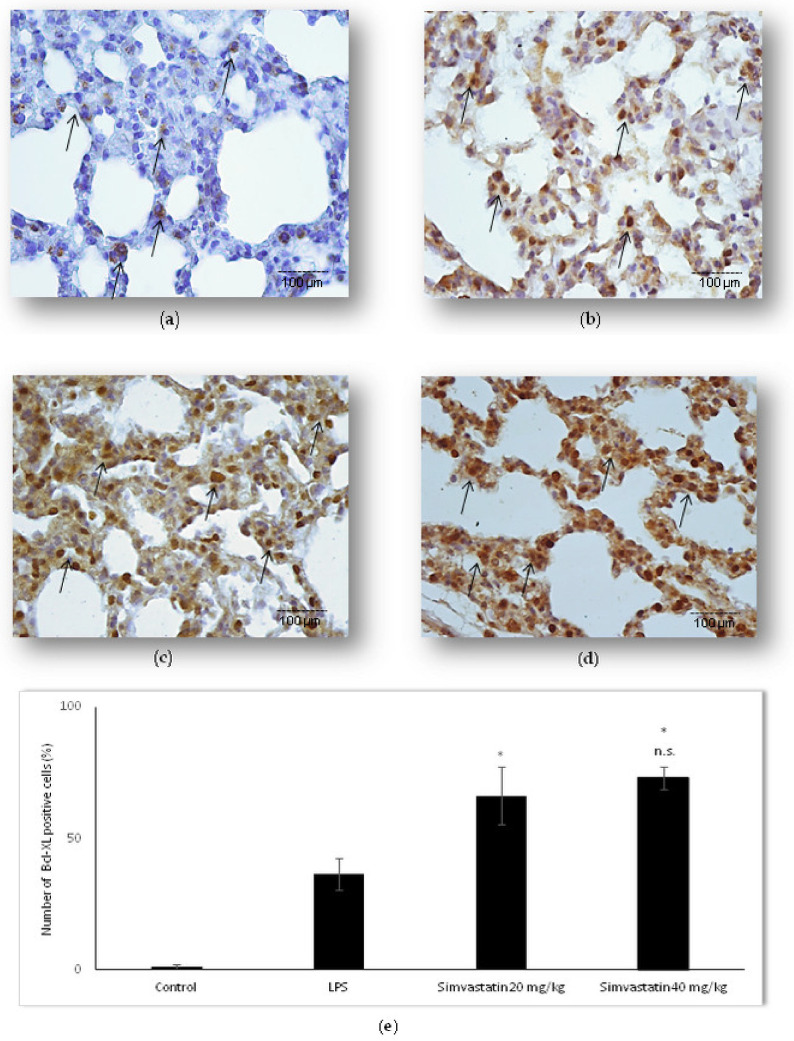
Simvastatin up-regulated Bcl-xL expression in alveolar epithelial cells in LPS-induced inflammation. Representative images of pulmonary parenchyma that were challenged with LPS or pretreated with 20 mg/kg of simvastatin or 40 mg/kg of simvastatin, respectively, priot to LPS, assessed by immunohistochemistry (400×). (**a**) Control group. (**b**) Numerous Bcl-xL-positive alveolar epithelial cells in the LPS group, in the 20 mg/kg of simvastatin (**c**) and in the 40 mg/kg of simvastatin group (**d**) Bcl-xL expression was significantly intensive in alveolar epithelial cells and macrophages (arrows). (**e**) Semiquantitative analysis of distribution of Bcl-xL positive cells in selected fields, * *p* < 0.05 in comparison to the LPS group, n.s. in comparison to 20 mg/kg of simvastatin.

**Figure 7:**
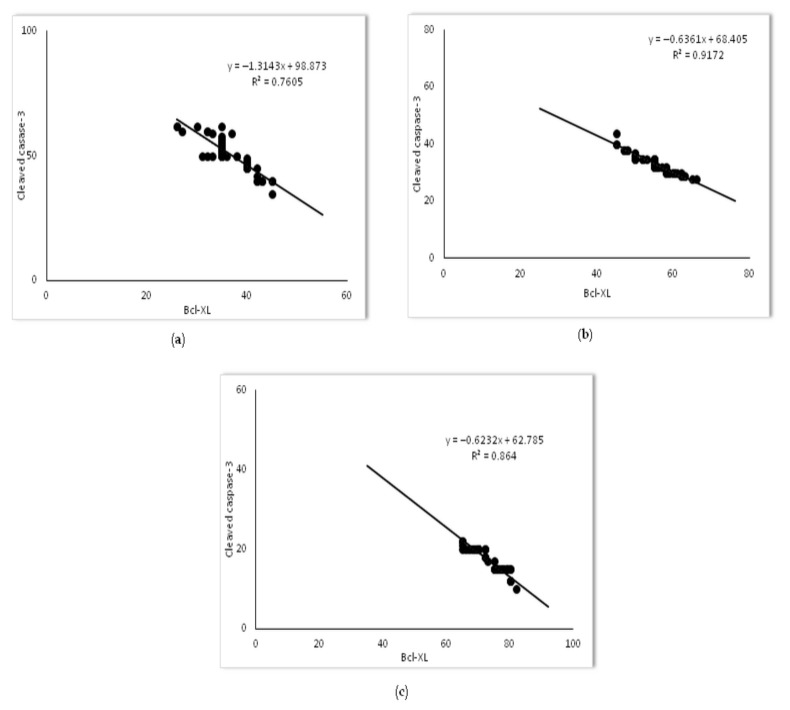
The correlations for Bcl-xL and cleaved-caspase-3 in alveolar epithelial cells are shown: (**a**) LPS group, (**b**) 20 mg/kg of simvastatin, (**c**) 40 mg/kg of simvastatin. The number of Bcl-xL and cleaved caspase-3 immunopositive alveolar epithelial cells was quantified across ten non-successive fields per sample of lung tissue from the LPS, 20 mg/kg of simvastatin, and 40 mg/kg of simvastatin groups, using ImageJ software 1.50. The correlation analysis and level of significance were calculated by using Pearson’s correlation coefficient.

The balance of anti-apoptotic Bcl-xL to pro-apoptotic proteins, such as cytochrome C and cleaved caspase-3, is an important determinant of mitochondrial membrane integrity, preventing cytochrome C release into the cytosol. We were interested in looking at Bcl-xL expression relative to pro-apoptotic proteins in LPS-induced ALI as a potential mechanism of simvastatin anti-apoptotic effect. The results revealed weak expression of Bcl-xL in the control group (Figure 6a), but in contrast, a significant increase of the mean percentage of moderately immune-positive alveolar epithelial cells in the LPS group (*p* < 0.05) (Figure 6b). Pretreatment with 20 mg/kg of simvastatin as well as with 40 mg/kg of simvastatin resulted in significant up-regulation of Bcl-xL with intensive brown cytoplasmic staining in alveolar epithelial cells and macrophages relative to the LPS group (66.4 ± 10.8% and 73.1 ± 4.5%, *p* < 0.05, respectively) (Figure 6c–e). The results showed the strong inverse correlation between Bcl-xL and cleaved caspase-3-positive cells across the groups (R^2^ = 0.76, *p* < 0.01 in the LPS group, R^2^ = 0.91, *p* < 0.01 in the 20 mg/kg of simvastatin group, and R^2^ = 0.86, *p* < 0.01 in the 40 mg/kg of simvastatin group) (Figure 7). Similarly, Bcl-xL expression was in a strong negative correlation with cytochrome C in the groups pretreated with 20 mg/kg of simvastatin and 40 mg/kg of simvastatin (R^2^ = 0.76, and R^2^ = 0.80, *p* < 0.01, respectively), suggesting that induction of Bcl-xL might be the potential mechanism of simvastatin against LPS-induced apoptosis. 

### 2.4. Survivin Was Involved in Simvastatin-Ameliorated Apoptosis of Alveolar Epithelial Cells Induced by LPS

**Figure 8:**
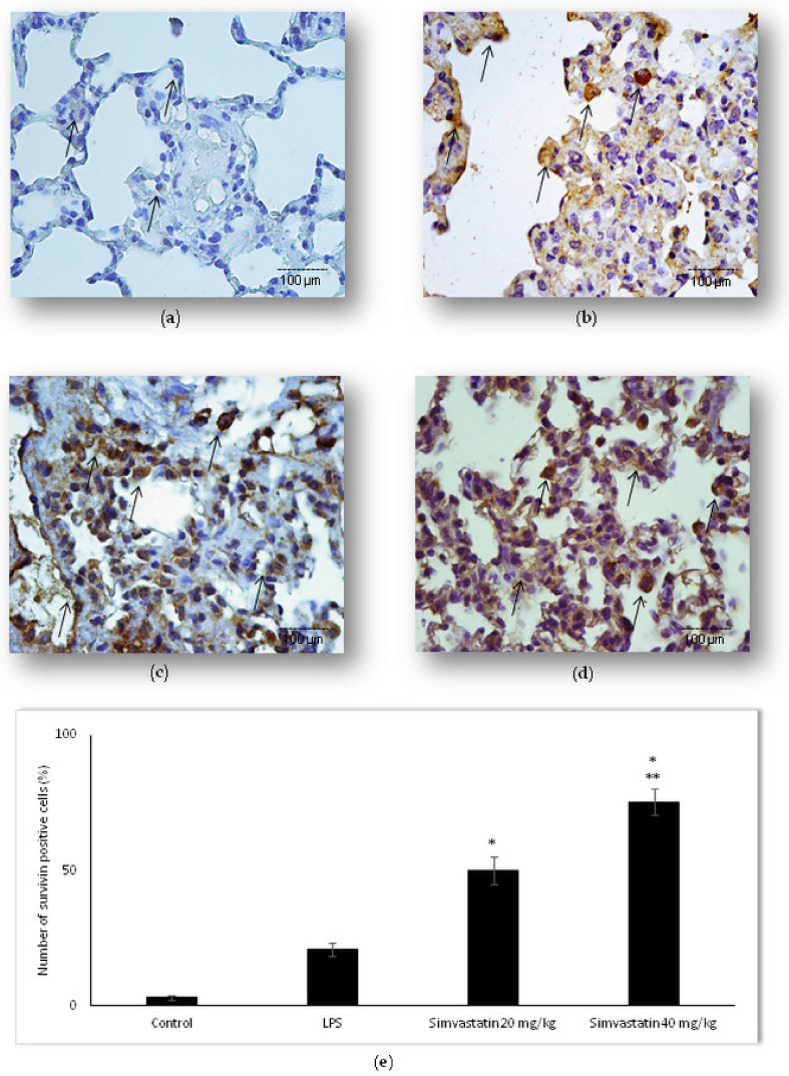
Survivin expression in alveolar epithelial cells was increased by simvastatin in LPS-induced inflammation. Representative images of pulmonary parenchyma that were challenged with LPS or pretreated with 20 mg/kg of simvastatin or 40 mg/kg of simvastatin, respectively, prior to LPS, assessed by immunohistochemistry (400×). (**a**) Control group. (**b**) LPS group. Markedly intensive cytoplasmic staining related to increased survivin expression (arrows) of alveolar epithelial cells and macrophages in the 20 mg/kg of simvastatin group (**c**) and 40 mg/kg of simvastatin group (**d**). (**e**) Semiquantitative analysis of distribution of survivin-positive cells in selected fields, * *p* < 0.01 vs. the LPS group, ** *p* < 0.01 vs. 20 mg/kg of simvastatin.

Following the findings that simvastatin enhanced Bcl-xL expression in pulmonary parenchyma after LPS administration, we further analyzed if simvastatin enhanced expression of survivin, a downstream inhibitor of apoptosis (Figure 8). Inflammation induced by LPS led to a significant increase of survivin expression (*p* < 0.05 vs. control) in alveolar epithelium and macrophages, indicating that this might present a cell-protective mechanism against LPS-induced injury (Figure 8b). The most important results demonstrate that simvastatin significantly and dose-dependently increased survivin expression shown as strong cytoplasmic staining in alveolar epithelial cells (Figure 8c,d). Semiquantitative analysis revealed that pretreatment with 20 mg/kg and 40 mg/kg of simvastatin, respectively, triggered marked increase of survivin expression (49.8% ± 5.0% and 75.4% ± 4.8% vs. LPS group, *p* < 0.01, respectively) (Figure 8e).

**Figure 9:**
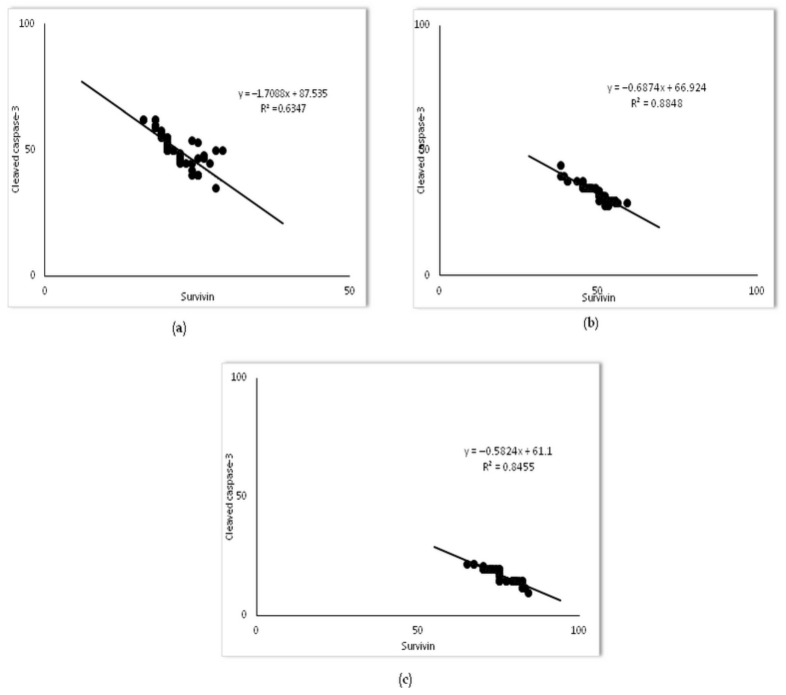
The correlations for survivin and cleaved-caspase-3 in alveolar epithelial cells are shown: (**a**) LPS group, (**b**) 20 mg/kg of simvastatin, (**c**) 40 mg/kg of simvastatin. The number of survivin and cleaved caspase-3 immunopositive alveolar epithelial cells was quantified across ten non-successive fields per sample of lung tissue from the LPS, 20 mg/kg of simvastatin, and 40 mg/kg of simvastatin groups, using ImageJ software 1.50. The correlation analysis and level of significance were calculated by using Pearson’s correlation coefficient.

Furthermore, Pearson’s correlation analysis revealed a strong inverse correlation of survivin with cleaved caspase-3-positive alveolar epithelial cells across each group (R^2^ = 0.63 in the LPS group, R^2^ = 0.88, in the 20 mg/kg of simvastatin group, and R^2^ = 0.84 in the 40 mg/kg of simvastatin group, *p* < 0.01, respectively) (Figure 9). These results indicate that simvastatin may inhibit LPS-induced apoptosis of alveolar epithelial cells through the activation of the cell-survival pathway, such as up-regulation of the inhibitor of apoptosis.

### 2.5. Changes of NF-kB Expression in Pulmonary Parenchyma after Simvastatin and LPS Administration

**Figure 10:**
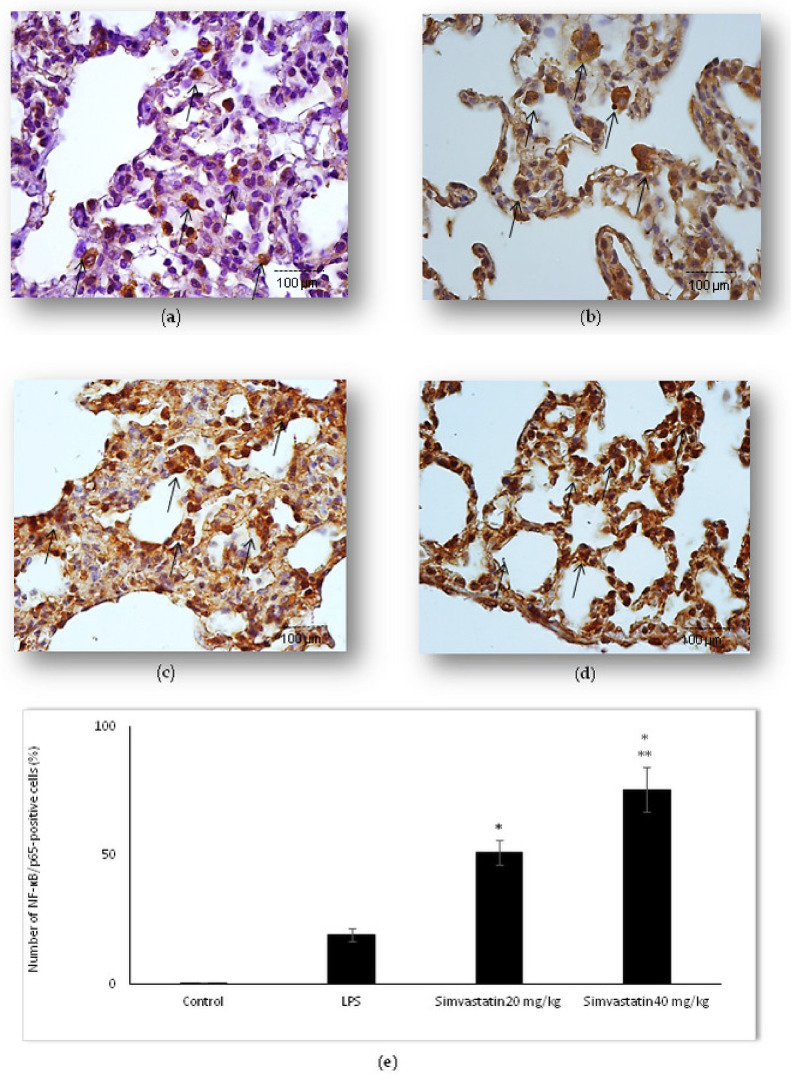
Simvastatin increased NF-kB/p65 expression in alveolar epithelial cells and macrophages in LPS-induced inflammation. Representative images of pulmonary parenchyma that were challenged with LPS or pretreated with 20 mg/kg of simvastatin or 40 mg/kg of simvastatin, respectively, prior to LPS, assessed by immunohistochemistry (400×). (**a**) Control group shows rare NF-kB/p65-positive cells. (**b**) In the LPS group, a significant number of alveolar epithelial cells and macrophages are positive for NF-kB/p65 in the cytoplasm and/or nucleus. Intensive nuclear staining indicates increased NF-kB/p65 expression (arrows) in alveolar epithelial cells and macrophages in the 20 mg/kg of simvastatin (**c**) and 40 mg/kg of simvastatin groups (**d**). (**e**) Semiquantitative analysis of NF-kB/p65-positive cells in selected fields, * *p* < 0.01 vs. the LPS group, ** *p* < 0.05 vs. 20 mg/kg of simvastatin.

Activation of NF-kB means translocation of p65 subunit from the cytoplasm to the nucleus. Basal expression NF-kB/p65 is shown as weak cytoplasmic staining of alveolar epithelial cells in the control group. Our results showed that LPS administration led to an increased number of NF-kB/p65-positive epithelial cells, including alveolar macrophages with intensive brown staining in the cytoplasm and/or nucleus (Figure 10b). Nuclear immunostaining of alveolar epithelial cells indicating activated NF-kB/p65 was significantly expressed in both groups treated with simvastatin (either 20 mg/kg of simvastatin or 40 mg/kg of simvastatin) in comparison to the group treated with LPS only (*p* < 0.01, respectively) (Figure 10c–e).

**Figure 11:**
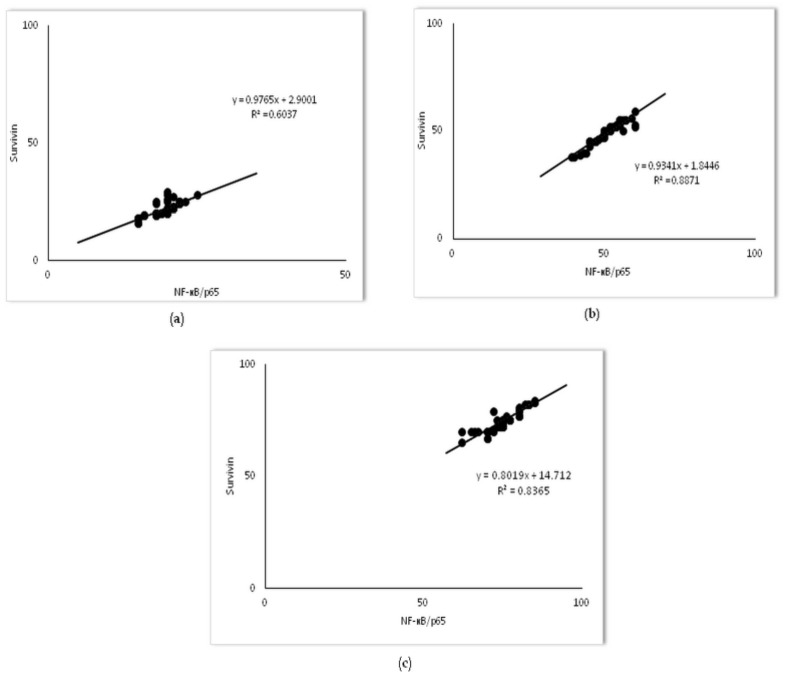
The correlations for survivin and NF-kB/p65 in alveolar epithelial cells are shown: (**a**) LPS group, (**b**) 20 mg/kg of simvastatin, (**c**) 40 mg/kg of simvastatin. The number of survivin and NF-kB/p65 immunopositive alveolar epithelial cells were quantified across ten non-successive fields per sample of lung tissue from the LPS, 20 mg/kg of simvastatin, and 40 mg/kg of simvastatin groups, using ImageJ software 1.50. The correlation analysis and level of significance were calculated by using Pearson’s correlation coefficient.

To test if the nuclear activity of NF-kB/p65 interplays with survivin expression in alveolar epithelial cells in LPS-induced inflammation, the Pearson correlation test was done. The results showed strong positive correlations between survivin and NF-kB/p65 expression across each group (R^2^ = 0.60 in the LPS group, R^2^ = 0.88, in the 20 mg/kg of simvastatin group, and R^2^ = 0.83 in the 40 mg/kg of simvastatin group, *p* < 0.01, respectively) (Figure 11).

## 3. Discussion

The major results obtained in sepsis-induced ALI showed that systemic exposure to LPS caused serious deterioration of pulmonary histology particularly alveolar epithelium, which were markedly and in a dose-dependent manner ameliorated by simvastatin. Endotoxin led to apoptosis mediated by cytochrome C and consequent cleavage of caspase-3 that was distributed in the complete pulmonary parenchyma, including alveolar epithelium and macrophages and endothelial cells. Following simvastatin administration, the extent of apoptosis induced by LPS was significantly reduced, and associated with up-regulated expression of anti-apoptotic Bcl-xL, IAP survivin, and transcriptional NF-kB/p65, mainly in alveolar epithelial cells. Taking the results above together with our previous comprehensive research [20,21,22], inhibition of apoptosis and activation of Bcl-xL and the survivin/NF-kB/p65 pathway seem to be the principal mechanisms of the protective effects of simvastatin in LPS-induced multi-organ dysfunction. 

Numerous studies investigating the therapeutic potential of statins in sepsis-induced ALI and ARDS patients have been conducted. A meta-analysis of 13 studies found that treatment with statins in patients with community-acquired pneumonia was associated with improved survival, particularly in those who were treated prior to hospital admission [23]. A randomized clinical study showed that prior statin use was associated with a lower baseline plasma IL-6 in patients with severe sepsis and improved 28-day survival, and the authors hypothesized that pretreatment with statins in critically ill patients at risk of sepsis may be more effective than treating established sepsis [24]. However, clinical trials with statins showed conflicting results in terms of improved clinical outcomes in patients with ARDS [25,26]. In contrast, in the animal model of sepsis-induced ALI, different statins attenuated inflammatory injury and apoptosis in pulmonary parenchyma, enhanced antioxidant enzymes, and improved endothelial permeability and the level of junction proteins through regulating different pathways, such as Cav-1/eNOS or TLR4/NF-kB [14,15,18,27]. Furthermore, the importance of a mevalonate pathway blockade that mimics statins’ mechanism resulted in more significant protection against LPS-induced ALI and alveolar epithelial apoptosis in lung-specific GGPPS1 knockout mice compared with simvastatin, which causes upstream inhibition of mevalonate pathway in cholesterol synthesis [8].

Current evidence suggests that dysregulated apoptosis of alveolar epithelium, endothelia, neutrophils, and alveolar macrophages may be potentially detrimental in septic ALI [1,2,3]. Caspase-3 activation is regulated by both TNF-α receptor-mediated extrinsic and intrinsic (mitochondrial-dependent) apoptosis cascades. In this regard, as we confirmed decreased overproduction of TNF-α in endotoxemia by simvastatin [19], this may explain reduced apoptosis in pulmonary parenchyma in the present study. Similar studies showed that pivastatin reduced apoptosis by decreasing caspase-3 expression and TUNEL-positive pulmonary cells in CLP-induced ALI through the activation of the phosphatidylinositol 3-kinase (PI3K)/Akt pathway, which is impaired under septic conditions [28]. 

The mitochondrial-dependent apoptosis is activated through the release of cytochrome C into the cytosol and consequent activation of caspase-3 and 7. This is controlled on the outer mitochondrial membrane by Bcl-2-related anti-apoptotic Bcl-xL that binds to pro-apoptotic Bim/Bax proteins and prevents the release of cytochrome C and apoptosis-inducing factors [29,30]. Our results suggested that LPS-induced up-regulation of the cytochrome C and down-regulation of the Bcl-xL in alveolar epithelial cells and macrophages were significantly improved by simvastatin. Accordingly, the anti-apoptotic mechanism of different statins through increased Bcl-xL or change of Bcl-2/Bax ratio have been confirmed in renal tubular cells [22], as in ALI induced by CLP [15,18], or hyperbaric oxygen [31]. 

The dual role of survivin has been associated with different cell compartments. Nuclear survivin facilitates cell proliferation, while cytoplasmic (mitochondrial) improves stability against apoptosis by inhibiting activation of effector caspases [10,32]. Previous studies demonstrated that cytoplasmic survivin was up-regulated in epithelial cells of bronchioles and alveoli in ALI induced by LPS or other agents [11,12], and its level was decreased with damage resolution [33], suggesting that survivin triggers cell protection similarly to LPS. Our study demonstrated that simvastatin enhanced cytoplasmic survivin expression in contrast to decreased cleaved caspase-3 and apoptosis of alveolar epithelial cells. Furthermore, the present study showed that nuclear expression of NF-kB/p65 was markedly increased by simvastatin in pulmonary cells, which showed an insignificant correlation with survivin expression. NF-kB has a vital role in inflammation, immune response, control of apoptosis, and cell survival, and is associated with the transcriptional up-regulation of survivin [10]. Cytoprotection by survivin, in part, targets the cascade of cytochrome C-mediated apoptosis to prevent downstream caspase-3 activation [6,32]. Wilson et al. [34], similarly to our previous work [20,21,22], have shown a significant role of survivin/NF-kB/p65 pathway activation in cytoprotection against sepsis-induced organ injuries. In contrast, considering of various pleiotropic effects the statins are likely to have anticancer effects (such as inhibition of proliferation and metastasis of malignant cells) by influencing inflammatory- and oxidative stress-related tumorigenesis [16,17].

Thus, to our best knowledge, it is most likely the first study that demonstrated the antiapoptotic action of simvastatin through up-regulation of survivin/NF-kB/p65 pathway as one of the principal mechanisms by which statin ameliorates septic ALI. However, further experimental research is warranted, particularly with respect to sepsis induced by infection, duration of the treatment, and the effects of simvastatin treatment on alveolar epithelial barrier histology and cell-survival after injury or infection have been sustained. In this regard, clinical studies should be focused on the clinical outcomes and simvastatin safety in the patients observed in several cohorts: statin-naive, those who are critically ill and at high risk of developing sepsis who would be pretreated with statins, and those with established sepsis who are preexisting statin users or who will be treated with a statin after the diagnosis is confirmed.

Even though simvastatin was administered in the animal model of ALI equivalently to clinical practice, our research has some limitations. First, LPS-induced ALI is a secondary response related to systemic inflammation caused by endotoxin only, rather than to bacteremia itself, such as in the CLP model. To strengthen the results of a protective effect of simvastatin in ALI, this will be evaluated in other septic lung injury models. Second, simvastatin was given as pretreatment but not after LPS exposure, which is of more clinical relevance. Therefore, its protective effect after sepsis induction will be investigated in further experiments. Finally, only male rats were examined in this experiment, so the potential anti-inflammatory impact of estrogen in female animals is excluded. 

## 4. Materials and Methods

### 4.1. Animals

Experimental design, laboratory protocol, and animal welfare were approved by the Ethics Committee of Experimental Animals of the Military Medical Academy, Belgrade, Serbia (No. 282-12/2002) before the start of the experiment. This approval confirmed that throughout the experiments, animal care and administered agents were in compliance with Directive 2010/63/EU on the protection of animals used for scientific purposes and the Guidelines for Animal Welfare adopted by the Republic of Serbia (No. 323-07-04943/2014-05/1).

We used experimental animals, adult Wistar rats, 6–8 weeks old (200–220 g), raised at the Institute for Biomedical Researches, Military Medical Academy, Belgrade, Republic of Serbia. According to the laboratory protocol, the animals were housed in the housing room in typical macrolon plastic cages (Bioscape, Castrop-Rauxel, Germany) filled with clear sawdust (Versele-Laga, Deinze, Belgium), with a centrally regulated air temperature of 22 ± 2 °C, humidity of 55% ± 15%, air changes/h of 15–20, and a light/dark cycle of 12/12 h. The animals were fed a commercial diet mixture for rats (produced by Veterinary Institute Subotica, Subotica, Republic of Serbia) and tap water ad libitum was applied.

### 4.2. Pharmacological Agents

Aseptic conditions were in place in each invasive procedure in animals. Acute inflammation was induced by lipopolysaccharide (LPS, endotoxin, producer Sigma Aldrich, Munich, Germany), serotype 0127:B8 *Escherichia coli*, dissolved with sterile pyrogen-free physiologic saline. Lipopolysaccharide (LPS) was administered intraperitoneally (i.p.) immediately after dilution.

Simvastatin, the drug used as a treatment (donation for research purposes only, from pharmaceutical company Krka, Novo Mesto, Slovenia) was dissolved in 0.5% methylcellulose as 10 or 20 mg/mL stocks.

### 4.3. Experimental Protocol

Experimental sepsis was induced with a non-lethal single dose of LPS i.p. determined in our previous work as 0.25 of the mean lethal dose (LD_50_) LPS per kg [19]. Endotoxin-induced experimental sepsis is a widely accepted model of human disease that is featured with high-grade systemic inflammation, inflammatory infiltration, pro-inflammatory cytokines and chemokines production, tissue injuries including ALI, and apoptosis [2,3,19]. The doses of simvastatin were based on the previous rat/murine in vivo studies. It has been determined that a dose range of 10–100 mg/kg/day is equivalent to those used in clinical medicine considering a rapid up-regulation (3- to 10-fold) of HMG-CoA reductase’s activity induced by statin treatment or other inhibitors in rodents. Therefore, in this experiment, simvastatin doses were higher compared to those recommended in clinical medicines [19,35,36].

In our previous experiments, simvastatin was administered in three different doses (10, 20, and 40 mg/kg p.o.), and completely prevented lethal outcome in rats against the single median lethal dose (LD_50_) of 22.15 mg/kg i.p. of LPS (95% CI 16.5–29.1) [19].

This experimental protocol consisted of five experimental groups, which were treated with the following agents:

(1) Control group (0.5% methylcellulose 1 mL/kg i.p.);
(2) LPS group (non-lethal dose as 0.25 LD_50_/kg i.p., which is equal to 5.5 mg/kg of LPS i.p.);
(3) 10 mg/kg of simvastatin group (10 mg/kg of simvastatin p.o. + 0.25 LD_50_/kg LPS i.p.);
(4) 20 mg/kg of simvastatin group (20 mg/kg of simvastatin p.o. + 0.25 LD_50_/kg LPS i.p.);
(5) 40 mg/kg of simvastatin group (40 mg/kg of simvastatin p.o. + 0.25 LD_50_/kg LPS i.p.).

Simvastatin was given orally by oral gavage, once daily over 5 days as a short-term pretreatment. On the last day of the pretreatment, the single non-lethal dose of LPS was administered 1.5 h after the simvastatin. The control group received vehicle only, while animals in the LPS group received the same vehicle (1 mL/kg) of 0.5% methylcellulose for the same period as the simvastatin pretreatment, before LPS injection. All animals were sacrificed 48 h after LPS administration.

### 4.4. Histopathological Evaluation and Semi-Quantitative Analysis of Pulmonary Damage Score

All animals were immobilized in a dorsal position, euthanized by using sodium pentobarbital in a single dose of 30 mg/kg i.p. (Hemofarm AD, Vršac, Republic of Serbia), and prepared for further standard histopathological analysis. We excised the left lungs of rats from each animal and fixed them in 10% neutral solution for one week. Tissue dehydration was carried out by immersing specimens in a series of ethanol solutions of increasing concentration until pure, water-free alcohol is reached. A series of increasing concentrations was used to avoid excessive distortion of the tissue. Fixed tissue samples were divided into six equal sections, which were dehydrated in a series of alcohol (70%, 96%, and 100%) and xylene. After fitting into paraffin blocks, each 2 µm thick pulmonary tissue section was stained using the hematoxylin and eosin (H&E) method. For detailed histopathological and semiquantitative analyses, Olympus BKS-43 (Olympus Europa, Hamburg, Germany) with a digital camera and Cell D software (Olympus, Munster, Germany) were used. Complete analyses were performed by pathologists blind of the treatment groups as per our method previously published in the literature [20,21,22,37,38,39,40,41,42,43].

### 4.5. Semiquantitative Analyses

The intensity of inflammatory injury of the lungs was scored and counted in 30 tissue slices per group (i.e., five tissues from each experimental group and six slices from each tissue) with a magnification of 200×, in accordance with previously published literature [20,21,22,37,38,39,40,41,42,43]. The severity grades were expressed as a pulmonary damage score (PDS), quantified, and precisely calculated. To assess the degree of lung damages, which consists of edema, hyperemia, neutrophil infiltration, and hemorrhages, a semi-quantitative five-point scale was applied as previously described [20,21,22,37,38,39,40,41,42,43]. 

### 4.6. Detection and Quantification of Cell Apoptosis In Situ by the TUNEL Method

To assess cell apoptosis, we used the TUNEL (Terminal deoxynucleotidyl transferase-mediated dUTP Nick End Labeling) assay on paraffin-embedded sections of 4–6 µm thickness (In-Situ Cell Death Detection Kit POD, Roche Molecular Biochemicals, Basel, Switzerland, Cat. No 11 684 817 910) according to the manufacturer’s instructions. Briefly, lung tissue slides were incubated with an anti-fluorescein antibody conjugated with horseradish peroxidase (POD), and then, color development was performed using diaminobenzidine (DAB) substrate. Negative (incubation with Label Solution, instead of TUNEL reaction) and positive controls (incubation with DNase I recombinant, grade I) were also performed. Ten non-successive fields per sample were analyzed counted for the number of TUNEL-positive cells under a light microscope (Olympus Plaza, Tokyo, Japan) at a magnification of 400×. The degree of apoptosis extent was expressed as an apoptotic index (AI), defined as the percentage (%) of TUNEL-positive cells, and calculated using the following formula, according to previously published literature [20,21,22]:

AI (% of apoptotic cells) = the number of TUNEL − positive cells × 100/total number of cells.

### 4.7. Immunohistochemistry in the Detection of Apoptosis

Sections of lung tissues were stained with the following antibodies: cleaved caspase-3 (Asp 175, Cat. 9661, Cell Signaling Technology, Frankfurt, Germany), Cytochrome C, clone 7H8.2C12 (Cat. MA5-11674, Invitrogen, Thermo Fisher Scientific, Walthman, MA, USA), Bcl-xL (Cat. PA1-37161, Invitrogen, Thermo Fisher Scientific, Walthman, MA, USA), survivin, clone 8E2 (Cat. MS-1201-P1 NeoMarkers Inc., Fremont, CA, USA), and NF κB/p65 (RB-1638-R7 NeoMarkers Fremont, CA, USA), according to the manufacturer’s instructions. Following the standard protocol for the immunohistochemistry staining, 3–4 µm tissue sections were deparaffinized and rehydrated, and then the slides were boiled with a citric acid buffer solution (0.01 mol/L citrate buffer, pH 6.0.). Non-specific background staining was removed by incubation in 3% hydrogen peroxide. Primary antibodies for cleaved caspase-3 (1:300), Cytochrome C (1:100), Bcl-xL (RTU), and survivin (1:50) were applied according to the manufacturer’s instruction. As chromogen, 3,30-Diaminobenzidine (DAB) (TL-015-HDJ, Thermo Scientific Lab Vision UltraVision ONE Detection System) was used to develop the antigen-antibody complex. Finally, all slides were counterstained with H&E, dehydrated, mounted, and analyzed with a microscope (Olympus, Tokyo, Japan) at 400× magnification. 

Expression of a certain molecule was quantified by calculating the percentage (%) of immune-positive cells (alveolar epithelial cells with intensive optical density expression of cleaved caspase-3, Cytochrome C, Bcl-xL, and survivin) across ten non-successive fields, by two independent pathologists in a blinded manner, using ImageJ software 1.50 (National Institute of Health, Bethesda, Rockville, MD, USA). Survivin expression was considered immuno-positive, where alveolar epithelial cells showed brown cytoplasmic staining [10,11]. Cytoplasmic expression of NF-kB/p65 is detected in most normal cells, but only distinct brown nuclear immunostaining (brown granules in the nucleus) is considered as activated NF-kB/p65 and quantified as previously described [10,11,20,21,22,32,44]. The number of survivin or NF-kB/p65-positive alveolar epithelial cells was expressed as a percentage (%), using the formula:

Percentage of survivin or NF-kB/p65-positive stained cells (%) = the number of positively stained alveolar epithelial cells/total number of alveolar epithelial cells × 100

### 4.8. Statistical Analysis

The results were analyzed by the statistical software package SPSS 19.0. (IBM Corporation, New York, NY, USA). The pulmonary damage score and biomarkers’ expressions across the experimental groups were tested by Kruskal–Wallis rank test and analysis of variance (one-way ANOVA) followed by the Tamhane’s T2 post hoc test, respectively, and Pearson’s correlation coefficient was employed in the correlation analysis. Thus, *p* < 0.05 was considered statistically significant.

## 5. Conclusions

In summary, the data above suggested that simvastatin pretreatment ameliorates LPS-induced ALI by suppressing inflammatory injury and alveolar endothelial barrier damage. The potential protective mechanism of simvastatin in LPS-induced ALI is associated with inhibition of cytochrome C and caspase-3 mediated apoptosis of alveolar epithelial cells. These findings are associated with increased cell survival through Bcl-xL and survivin/NF-kB/p65 signaling pathway up-regulation. These data add important insights into the class of drugs known as statins and their protective, anti-inflammatory, and anti-apoptotic properties in LPS-induced multi-organ dysfunction syndrome, including ALI, but these potential therapeutic effects and safe use in established sepsis associated with bacterial infection warrant further preclinical and clinical research.

## Figures and Tables

**Table 1:** The effect of simvastatin on the degree of lung histopathology caused by LPS—Pulmonary Damage Score (PDS).

Treatment (mg/kg)	Pulmonary Damage Score (6 Lung/Group × 6 Slices/Lung)						
	0	1	2	3	4	5	$\bar{X}$ ± SD
Control	31	5	0	0	0	0	0.60 ± 0.11
LPS group	0	0	4	20	12	0	3.33 ± 0.48 ^a1^
10 mg/kg of simvastatin group	0	0	24	10	2	0	2.33 ± 0.48 ^a2^
20 mg/kg of simvastatin group	0	6	24	6	0	0	2.00 ± 0.59 ^a2,b1^
40 mg/kg of simvastatin group	0	23	13	0	0	0	1.33 ± 0.40 ^b1^

Kruskal–Wallis test was performed using the ^a1^ *p* < 0.001 in comparison to the control, ^a2^ *p* < 0.05 in comparison to the control, and ^b1^ *p* < 0.01 in comparison to the LPS group.

## Data Availability

Not applicable.
